# Policing of gut microbiota by the adaptive immune system

**DOI:** 10.1186/s12916-016-0573-y

**Published:** 2016-02-12

**Authors:** Laurent Dollé, Hao Q. Tran, Lucie Etienne-Mesmin, Benoit Chassaing

**Affiliations:** Laboratory of Liver Cell Biology, Department of Basic Biomedical Sciences, Faculty of Medicine and Pharmacy, Vrije Universiteit Brussel (VUB), Brussels, Belgium; Institute for Biomedical Sciences, Center for Inflammation, Immunity, and Infection, Georgia State University, Atlanta, GA 30303 USA

**Keywords:** Adaptive immunity, Immunoglobulin, Microbiota, Inflammation, Immunoglobulin-based therapy

## Abstract

The intestinal microbiota is a large and diverse microbial community that inhabits the intestine, containing about 100 trillion bacteria of 500-1000 distinct species that, collectively, provide benefits to the host. The human gut microbiota composition is determined by a myriad of factors, among them genetic and environmental, including diet and medication. The microbiota contributes to nutrient absorption and maturation of the immune system. As reciprocity, the host immune system plays a central role in shaping the composition and localization of the intestinal microbiota. Secretory immunoglobulins A (sIgAs), component of the adaptive immune system, are important player in the protection of epithelium, and are known to have an important impact on the regulation of microbiota composition. A recent study published in *Immunity* by Fransen and colleagues aimed to mechanistically decipher the interrelationship between sIgA and microbiota diversity/composition. This commentary will discuss these important new findings, as well as how future therapies can ultimately benefit from such discovery.

## Background

The gut host defense system comprises an array of mechanisms to keep the microbiota in check, maintaining an orderly beneficial relationship with the intestinal microbiota [1]. These mechanisms include the presence of multi-layered mucus structures, secretion of anti-microbial peptides and the secretion of sIgA. Additionally, the mucosal immune system has several means to sample and evaluate potential danger of microbiota-derived antigens, allowing production of specific antibodies to bacterial antigens that might compromise the host. Adaptive immunity in general, and sIgA in particular, is known to play a key role in microbiota composition. Here, we will discuss recent findings describing how IgAs population has an impact on microbiota diversity, and how they may provide therapeutic insights into diseases associated with dysbiosis [2].

### Immunoglobulin A-mediated modulation of the intestinal microbiota

**Fig. 1:**
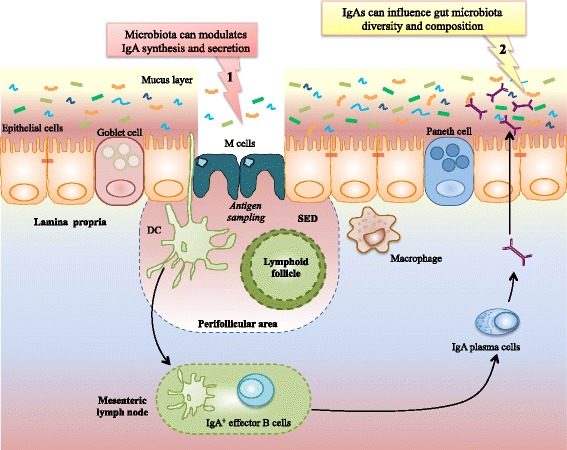
Interplay between IgA and the microbiota in the intestine. Schematic representation of IgA generation in the intestine, and how IgA population and intestinal microbiota regulate each other. Symbol 1: intestinal antigen sampling, mainly through M-cells process, is the first step in IgA plasma cells generation and IgA synthesis. IgA population and diversity will depend on antigenic peptides presented to the immune system by antigen-presenting cell. Symbol 2: after interaction with its receptor, IgA dimers are translocated to the lumen where they will provide mucosal immune protection. In addition, such secreted IgA can subsequently regulate microbiota composition, diversity, and gene expression. SED: sub-epithelial dome; DC: dendritic cell

A key intestinal strategy to generate immune protection in a non-inflammatory manner is the production of IgA [3–5], which is schematically illustrated in Fig. 1. One of the main role played by IgA is the promotion of immune exclusion by entrapping dietary antigens and microorganisms in the mucus, or down-regulating the expression of pro-inflammatory bacterial epitopes on commensal bacteria, such as flagellin [6]. IgA population in the gut is central for the selection and the maintenance of the intestinal microbiota [7, 8].

The main observation showing the importance of immunoglobulin in microbiota composition regulation was made with animals lacking such Ig production. Microbiota analysis of RAG1^-/-^ mice (which have no adaptive immune system owing to the lack of V(D)J recombination-activating protein 1 (RAG1)) revealed profound alterations in their microbiota composition [9]. Moreover, it was observed that the restoration of normal IgA levels in AID (activation-induced cytidine deaminase) deficient mice, which normally lack IgAs, is sufficient to restore a normal microbiota composition [7, 10, 11]. Another important observation showing that intestinal IgA help to shape the intestinal microbiota is the recent finding that cessation of breast-feeding to either formula or food drives the maturation of the infant gut microbiome, indicating that the important amount of IgA secreted in mother’s milk seems to play a central role in the regulation of microbiota composition [12]. Mounting evidence clearly reveals that bacterial species colonizing the gut differ in their ability to stimulate the post-natal maturation of the gut system, with a good example being segmented filamentous bacteria (SFB) which are potent stimuli of IgA responses and strong inducers of Peyer’s patches development [13, 14].

### Polyreactive IgA diversity controls microbiota composition and diversity

Fransen and collaborators recently demonstrated that the abundance and repertoire diversity of innate IgAs (also referred to as polyreactive due to their ability to bind multiple antigens) play a central role in regulating the diversity of the intestinal microbiota [2, 15]. The authors observed that C57BL/6 and BALB/c mice differ drastically in their IgA abundance and repertoire richness, which associate with profound differences in their microbiota composition. While BALB/c mice have a high abundance and diversity of IgAs, C57BL/6 mice harbor a poor IgA repertoire correlating with a decreased microbiota diversity. Even under germfree conditions (germfree animals are devoid of any microorganisms), C57BL/6 and BALB/c mice differ in polyreactive IgA, revealing a genetic component of IgA production. Importantly, those polyreactive IgA were found by the authors to determinate the capacity of the strain of mice to diversify the microbiota. Delving deeper into the mechanisms of such interrelationship between polyreactive IgA and microbiota diversity, the authors demonstrated that polyreactive IgAs are required to trigger IgA response toward members of the intestinal microbiota, through a coating that favor bacterial penetration into Peyer’s patches [2]. Those findings further demonstrate that microbiota diversity is both genetically and environmentally driven, mechanistically supporting earlier observations [16].

However, it is important to note that low IgA diversity is not the exclusive discrepancy found between these 2 mice strains. Among other differences, C57BL/6 mice have a normal Th1 response while BALB/c mice are deficient in Th1 signaling, which could also have an important impact on the phenotypes described in this study. One important point that still needs to be addressed is whether the decreased microbiota diversity observed in C57BL/6 mice, as a result of low IgA diversity, is associated with an increased pro-inflammatory potential/harmfulness. The investigations of whether the altered microbiota associated with low intestinal IgA predispose mice of subsequent challenges still need to be conducted.

### Immunoglobulin-based therapy for microbiota composition alteration and restoration?

It is important to note that, in the study by Fransen and colleagues, co-housing or fecal transplantation had little effect on both IgA production and microbiota composition, highlighting the stability of the intestinal microbiota in an individual, with a central role played by IgA repertoire [2]. The defined repertoire of IgAs controls the colonization and composition of the microbiota and will lead to the restoration of the original microbiota following alteration. Consequently, such findings suggest that modulation of IgA repertoire could lead to a more drastic and stable alteration in the intestinal microbiota compared to, for example, fecal transplantation. Moreover, it has been observed that mice bred in different facilities harbor a distinct microbiota that further determines the levels of secretory IgA. This study demonstrates that transfer of a microbiota from an IgA-low mice, by co-housing or fecal transplantation, can lower fecal IgA levels in IgA-high mice [17]. This study also shows that IgA-low mice are more susceptible to challenges such as Dextran Sulfate Sodium (DSS)-induced colitis, and that such susceptibility can be transferred to IgA-high mice by fecal transplantation and are driven by fecal IgA differences *via* a mechanism involving the ability of bacteria from IgA-low mice to degrade sIgA [17]. Altogether, those findings highlight the close relationship occurring between sIgA repertoire and the microbiota, with a key role played in the maintenance of intestinal homeostasis.

A common feature of colitis-associated microbiota are increased levels of bioactive flagellin and lipopolysaccharide (LPS), which can activate Toll-like receptor 5 (TLR5), NOD-like Receptor 4 (NLRC4) inflammasome, and TLR4 [18–20]. Approaches to manipulate the microbiota to make it inherently less pro-inflammatory (i.e. reduce levels of innate immune activators) may ultimately provide a novel approach to prevent and/or treat Inflammatory Bowel Disease (IBD). Published observations demonstrating that the level of microbiota flagellin expression inversely correlates with levels of fecal anti-flagellin antibodies suggests that the adaptive immune system possess the ability to alter the microbiota to make it less pro-inflammatory (Fig. 1) [21, 22]. Indeed, in a study published in *Cell Host and Microbes* in 2013, it was demonstrated that TLR5^-/-^ mice harbor a reduced level of flagellin specific IgA [21]. Importantly, the intestinal microbiota of those TLR5^-/-^ animals was found to express significantly higher amounts of bioactive flagellin, supporting an impact of intestinal IgA in suppressing levels of flagellin, likely by putting flagellated bacteria at a competitive disadvantage within a complex microbial community.

In addition, recent findings made by flow-cytometric sorting suggest that IgA may mark commensal and pathobionts according to the extent of their individual coating [23]. This study by Palm and colleagues show that IgA coating selectively marks known disease-driving members of the mouse and human intestinal microbiota that can impact disease susceptibility and/or severity [23]. Transfer of fecal IgA-coated from cohorts of Kwashiorkor undernourished children into germ free mice triggers a diet-dependent enteropathy with intestinal inflammation and dysfunction, but could be prevented by administering two IgA-targeted bacterial species from a healthy microbiota (Clostridium scindens, Akkermansia muciniphila) [24]. A targeted elimination or replacement of disease-driving members of the intestinal microbiota could be a first step in the development of personalized, microbiota reshaping therapies.

## Conclusions

Based on this appealing work by Rescigno and colleagues, we can hypothesis that selected manipulation of the immune system has the potential to alter gut microbiota composition to make it inherently less pro-inflammatory (i.e. more diverse and with a reduced level of innate immune activators), reducing susceptibility to and/or severity of intestinal inflammation development. IgA may be used as a target to shape the intestinal bacterial community in order to maintain a beneficial relationship between the host and the microbiota.

## Abbreviations

AID: Activation-induced cytidine deaminase
CD: Crohn’s disease
DSS: Dextran sodium sulfate
IBD: Inflammatory bowel disease
IgA: Immunoglobulin A
LPS: Lipopolysaccharide
NLRC4: Nod-like receptor C4
RAG1: Recombination-activating protein 1
SFB: Segmented filamentous bacteria
TLR: Tool-like receptor
UC: Ulcerative colitis
